# Supplementary material for: Identity-based linear homomorphic signature for a restricted combiners’ group for e-commerce
Source: PeerJ Comput Sci. 2025 Aug 4;11:e3068. doi: 10.7717/peerj-cs.3068 (PMC12453756; doi:10.7717/peerj-cs.3068)
Supplement: Supplemental Information 3 [file peerj-cs-11-3068-s003.pdf]

Confidentiality Proof

In this section, we will prove the confidentiality of the scheme under adaptive chosen message attacks in the random oracle model. We use the challenge-response mode for the proof. Let the challenger be denoted as $\mathcal{C}$, the adversary as $\mathcal{A}$, and the number of queries to the random oracle as q.

Initialization. The challenger $\mathcal{C}$ initializes the public parameters and master secret key as described in the initialization phase.

Random Oracle Queries. The adversary $\mathcal{A}$ adaptively queries the random oracle O_H. The challenger responds to these queries as follows:

- For each query $H(m)$, if m is already in the query table, return the stored value. Otherwise, randomly select a value from $\mathbb{G}_1$ and return it, storing the result in the query table.
- For each query to the vector value, if the vector is already in the query table, return the stored value. Otherwise, randomly select a value and return it, storing the result in the query table.
- For each query to the session key, if the session key is already in the query table, return the stored value. Otherwise, randomly select a value and return it, storing the result in the query table.

Signature Queries. The adversary $\mathcal{A}$ can request signatures on chosen messages. The challenger responds by generating the signatures as described in the signature phase.

Output Phase. The adversary $\mathcal{A}$ outputs a guess for the ID associated with the message. The challenger $\mathcal{C}$ checks if the guess is correct. If it is, the adversary wins the game.

The probability of the adversary $\mathcal{A}$ winning the game is analyzed as follows:

- Let A be the event that $\mathcal{A}$ wins the game.
- Let B be the event that the $\mathcal{A}$'s query is orthogonal to the challenge.

The probability of the adversary winning the game is given by:

$$Pr(A) = Pr(A|B) \cdot Pr(B) + Pr(A|\bar{B}) \cdot Pr(\bar{B}) \tag{1}$$

Since $Pr(B)$ is negligible, and $Pr(A|\bar{B})$ is also negligible, we can conclude that $Pr(A)$ is negligible. Thus, the scheme is confidential under adaptive chosen message attacks in the random oracle model.

Coalition-resistance Proof

Phase 1: Initialization and User Registration

- **Challenger's Operations**:
  - Generates public parameters $params$ and master private key $msk = s$.
  - For each user identity ID_i requested by the adversary, generates private key $usk_{ID_i} = (x_i, R_i)$, where $x_i = r_i + s \cdot H_0(ID_i, R_i)$ and $R_i = h^{r_i}$.
- **Collusion Resistance Analysis**:
  - Even if the adversary obtains multiple users' private keys $\{usk_{ID_i}\}$, they cannot recover s through linear combinations due to the independence of r_i and the secrecy of s (relies on the hardness of the discrete logarithm problem).

Phase 2: Combiner Permission Application

- **Challenger's Operations**:
  - Generates session keys $b_{ID} = B \mod q_{ID}$ for legitimate combiners and distributes them via CRT.
- **Collusion Resistance Analysis**:
  - If the adversary controls t' combiners with $\{b_{ID_j}, q_{ID_j}\}_{j=1}^{t'}$ and $t' < t$ (threshold), B cannot be recovered (CRT requires at least t pairwise coprime q_{ID}).
  - If the adversary forges q_{ID} or b_{ID}, the verification in Phase 5 detects $B' \neq B$ and terminates the protocol.

Phase 3: Signature Generation and Distribution

- **Challenger's Operations**:
  - Generates signatures $\sigma_k = \widehat{\sigma}_k^x \cdot H_3(v_k, ID)^{B \mod Q}$ for data $data$.
- **Collusion Resistance Analysis**:
  - To forge a signature, the adversary must compromise both x and B:
    - x is protected by user private keys and bound to s.
    - B is distributed via CRT; partial knowledge of b_{ID} is insufficient for recovery (relies on CRT security).

Phase 4: Verification and Combination

- **Challenger's Operations**:
  - Verifies $e(\varsigma_k, h) = e(\widehat{\sigma}_k, R \cdot mpk^{H_0(ID,R)})$ during validation.
- **Collusion Resistance Analysis**:
  - If colluders tamper with σ_k or $\widetilde{v}_d$, the bilinear pairing check fails (relies on collision resistance of hash functions and properties of bilinear maps).

Assume an adversary A can break collusion resistance with non-negligible probability ϵ. We construct an algorithm C' to solve the CDH problem:

1. C' simulates the scheme using A's queries and embeds a CDH instance into public parameters.
2. When A forges a signature, C' extracts the CDH solution from the bilinear map result.
3. By the CDH assumption, ϵ is negligible, leading to a contradiction. Hence, collusion resistance holds.

The scheme achieves collusion resistance through:

- **Master Key Protection**: Distributed generation and verification of s and B, preventing single-point leakage.
- **Session Key Distribution**: Threshold mechanism based on CRT, requiring colluders to exceed the security threshold.
- **Cryptographic Primitives**: Bilinear map verification and collision-resistant hash functions ensure tamper detection.
- **Dynamic Binding**: Signatures are bound to file identifiers τ, preventing replay attacks.

Thus, under the challenge-response model, the scheme resists collusion attacks.
