# Supplementary material for: Identity-based linear homomorphic signature for a restricted combiners’ group for e-commerce
Source: PeerJ Comput Sci. 2025 Aug 4;11:e3068. doi: 10.7717/peerj-cs.3068 (PMC12453756; doi:10.7717/peerj-cs.3068)
Supplement: Supplemental Information 4 [file peerj-cs-11-3068-s004.pdf]

Unlinkability Proof

Game Definition

1. **Setup**: The challenger generates $params$ and msk, and registers two users U_0 and U_1 with private keys usk_{ID_0} and usk_{ID_1}.

2. **Challenge Phase**: The challenger randomly selects $b \in \{0, 1\}$, uses usk_{ID_b} to generate a signature σ^*, and sends σ^* to the adversary.

3. **Adversary Queries**: The adversary can request signatures for other messages or users (excluding U_0 and U_1) and perform verifications.

4. **Guess**: The adversary outputs a guess b'. The scheme is unlinkable if:

$$\left|\Pr[b' = b] - \frac{1}{2}\right| \leq \mathsf{negl}(\lambda).$$

Key Observations

- **Private Key Randomness**: Each user's $usk_{ID} = (x, R)$ includes a unique random r in $R = h^r$. Since r is fresh per user, $x = r + s \cdot H_0(ID, R)$ is statistically independent across users.

- **Signature Randomization**: Signatures $\sigma_k = \widehat{\sigma}_k^x \cdot H_3(v_k, ID)^{B \mod Q}$ depend on both x (user-specific) and $B \mod Q$ (session-specific). The term $H_3(v_k, ID)^{B \mod Q}$ introduces session randomness, preventing linkage across different signatures.

- **Session Key Obfuscation**: The CRT-based distribution of $b_{ID} = B \mod q_{ID}$ ensures that partial knowledge of $\{b_{ID_j}\}$ does not reveal B unless t combiners collude. This threshold mechanism hides user-specific contributions.

Formal Reduction

Assume an adversary $\mathcal{A}$ can win the unlinkability game with non-negligible advantage ϵ. We construct a solver $\mathcal{S}$ for the CDH problem:

1. $\mathcal{S}$ embeds a CDH instance (g, h, g^a, h^b) into the public parameters and simulates user keys using a, b.

2. When $\mathcal{A}$ requests a signature, $\mathcal{S}$ programs the hash oracles to align with the CDH challenge.

3. If $\mathcal{A}$ successfully links signatures, $\mathcal{S}$ extracts $e(g, h)^{ab}$ from the bilinear pairing results, solving CDH.

4. By the CDH assumption, ϵ must be negligible, contradicting $\mathcal{A}$'s advantage. Hence, unlinkability holds.

Critical Analysis

- **Leakage Prevention**: No phase reveals s, B, or deterministic relationships between users' operations. The use of fresh randomness (r, B) in key generation and signing ensures unlinkability.

- **Verification Anonymity**: The verification equation $e(\varsigma_k, h) = e(\widehat{\sigma}_k, R \cdot mpk^{H_0(ID,R)})$ depends only on public values (R, mpk) and session-specific terms, avoiding user identity exposure.

- **Threshold Security**: The requirement of t combiners to recover B ensures that fewer colluders cannot compromise session anonymity.

The scheme achieves unlinkability through:

- Randomized private key generation and session-specific parameters.

- Threshold-based session key distribution via CRT.

- Cryptographic primitives (bilinear maps, collision-resistant hashes) that prevent leakage of user-specific information.

- Dynamic binding of signatures to session-specific terms (e.g., $B \mod Q$) rather than user identities.

Under the challenge-response model, the adversary cannot distinguish signatures from different users beyond random guessing, proving the scheme's unlinkability.

Traceability Proof

Game Definition

1. **Setup**: The challenger generates $params$, msk, and registers a set of users $\mathcal{U}$. Each user U_i receives $usk_{ID_i} = (x_i, R_i)$.

2. **Adversary Queries**: The adversary can:

   - Request user private keys for any $ID_j \in \mathcal{U}$.
   - Request signatures on messages with specified ID_j.
   - Corrupt combiners to obtain their session keys $\{b_{ID_j}\}$.

3. **Challenge**: The adversary outputs a forged signature σ^* on a message m^*, claiming it cannot be traced to any registered user.

4. **Tracing**: The challenger uses the tracing algorithm to extract an identity ID^* from σ^*. The scheme is traceable if:

$$\Pr\left[ID^* \in \mathcal{U} \wedge \text{Verify}(m^*, \sigma^*) = 1\right] \geq 1 - \mathsf{negl}(\lambda).$$

Key Mechanisms for Traceability

- **Identity Binding in Private Keys**: Each user's $usk_{ID} = (x, R)$ is bound to ID via $x = r + s \cdot H_0(ID, R)$. The term $H_0(ID, R)$ ensures that x uniquely encodes ID, and any valid signature must use a valid x linked to a registered identity.

- **Signature Structure**: Signatures $\sigma_k = \widehat{\sigma}_k^x \cdot H_3(v_k, ID)^{B \mod Q}$ explicitly include ID in H_3. During verification, the challenger can check the consistency of ID with the public parameters and traced keys.

- **Session Key Recovery via CRT**: The threshold-based recovery of B requires at least t honest combiners. If a forged signature uses an invalid B, the tracing algorithm can identify corrupt combiners by analyzing inconsistencies in B'.

Formal Reduction

Assume an adversary $\mathcal{A}$ can forge an untraceable signature with non-negligible probability ϵ. We construct a solver $\mathcal{S}$ for the DLP in G_1:

1. $\mathcal{S}$ simulates the scheme and embeds a DLP instance $h = g^a$ into the public parameters.

2. When $\mathcal{A}$ requests a signature for ID_j, $\mathcal{S}$ programs $H_0(ID_j, R_j)$ to align with the DLP challenge.

3. If $\mathcal{A}$ outputs a forged σ^*, $\mathcal{S}$ extracts x^* from $\widehat{\sigma}_k$ via:

$$e(\widehat{\sigma}_k, h) = e(g^{x^*}, h) \implies x^* = \log_g \widehat{\sigma}_k.$$

   Since $x^* = r + s \cdot H_0(ID^*, R^*)$, $\mathcal{S}$ solves a from $h = g^a$ using the extracted x^*.

4. By the DLP assumption, ϵ must be negligible, contradicting $\mathcal{A}$'s success. Hence, traceability holds.

Critical Analysis

- **Non-Frameability**: Even if the adversary corrupts users, they cannot forge signatures for honest users because x_i depends on s (unknown to the adversary).
- **Threshold Security**: The CRT-based recovery of B ensures that corrupting fewer than t combiners does not compromise B, preventing fake session keys from being accepted.
- **Public Verifiability**: The verification equation $e(\varsigma_k, h) = e(\widehat{\sigma}_k, R \cdot mpk^{H_0(ID,R)})$ ensures that only valid ID-bound signatures pass verification.

The scheme achieves traceability through:

- Cryptographic binding of user identities to private keys via $H_0(ID, R)$.
- Explicit inclusion of ID in signature components and hash functions.
- Threshold mechanisms for session key recovery, limiting collusion impact.

Under the challenge-response model, any forged signature can be traced to a registered user with overwhelming probability, proving the scheme's traceability.
